# Impact of organic acid cross-linking on the structure and functional properties of gum arabic and guar gum: Formulation of an edible coating for enhancing strawberry shelf life

**DOI:** 10.1016/j.fochx.2025.102527

**Published:** 2025-05-05

**Authors:** Anchal Choudhary, Mansuri M. Tosif, Aarti Bains, Gulden Goksen, Rupak Nagraik, Sanju Bala Dhull, Nemat Ali, Nazish Muzaffar, Prince Chawla

**Affiliations:** a Department of Food Technology and Nutrition, Lovely Professional University, Phagwara, Punjab 144411, India; b Department of Microbiology, Lovely Professional University, Phagwara, Punjab 144411, India; c Department of Food Technology, Vocational School of Technical Sciences at Mersin Tarsus Organized Industrial Zone, Tarsus University, 33100 Mersin, Turkey; d Department of Biotechnology, Graphic Era, Deemed to be University, 248002 Dehradun, India; e Department of Food Science and Technology, Chaudhary Devi Lal University, Sirsa 125055, Haryana, India; f Department of Pharmacology and Toxicology, College of Pharmacy, King Saud University, P.O. Box 2457, Riyadh 11451, Saudi Arabia; g College of Food Science and Technology, Henan University of Technology, Zhengzhou, China

**Keywords:** Polysaccharide, Gums, Organic acids: guar gum, Citric acid

## Abstract

This study investigates the impact of organic acid modification on the structural characteristics and functional properties of gum arabic (GA) and guar gum (GG) by incorporating different ratios of organic acids including citric acid (CA), malic acid (MA), and tartaric acids (TA). Results revealed that organic acid modification of GA resulted in an increase in particle size with higher organic acid concentrations (1:2 *w*/w ratio compared to 1:1 *w*/w). Moreover, GA modified by CA exhibited the highest DS values from 0.79 ± 0.04 to 0.81 ± 0.09. SEM analysis revealed uniform particles with noticeable agglomeration, while FTIR confirmed successful carboxyl group incorporation and ester bond formation, contributing to enhanced structural complexity. TGA indicated improved thermal stability. Furthermore, application on strawberries revealed that the modified gum coating effectively maintained freshness of strawberries over 16 days of storage. Overall, these findings revealed the potential of organic acid-modified gums to enhance their functional properties.

## Highlights

• Organic acids enhanced the degree of substitution and solubility of gum arabic (GA) and guar gum (GG).
• Citric acid (CA) contributed to the improvement of the techno-functional properties of gum.
• Spray-dried organic acid modified gum powder exhibited nano-scaled particle size.
• CA modification of GA improved the rheology of solution and potentially extended the shelf-life of strawberry.

## 1 Introduction

Gums are edible polysaccharides, that can be obtained from different sources and are characterized by their suitable structure and functional characteristics (Benalaya, Alves, Lopes, and Silva, 2024). The term “gum” refers to either a group of hydrophilic or hydrophobic polysaccharides with high molecular weight or polysaccharide derivatives capable of forming gels or viscous solutions in certain solvents at low concentrations (Thombare, Jha, Mishra, and Siddiqui, 2016). Chemically, the primary constituents of gums are typically galactomannans or glucomannans, wherein the linear units of -D-mannose and -d-glucose or -D-galactose are linked by −1 → 4 bonds, respectively (Doublier, Garnier, and Cuvelier, 2017). These polysaccharide gums, also known as hydrocolloids, are extensively utilized across diverse industries, including food, medical, textile, water, energy, biotechnology, environment, cosmetics, and pharmaceuticals due to their remarkable functional properties (Pirsa & Hafezi, 2023). Additionally, their ability to reduce surface tension enables the stabilization of different phases through spatial, electrostatic, and hydration force interactions. Gum Arabic, also known as acacia gum, is a natural gum derived from the Acacia trees, predominantly *Acacia Senegal* and *Acacia seyal*. It is a complex mixture of polysaccharides and glycoproteins (Ashour et al., 2022). Gum Arabic is soluble in water and forms a viscous solution, making it valuable in various industries such as food, pharmaceuticals, and cosmetics. Its main constituents are arabinogalactans, which are branched polysaccharides composed mainly of arabinose and galactose units (Prasad, Thombare, Sharma, and Kumar, 2022). Gum Arabic is widely used as an emulsifier, stabilizer, and thickener due to its excellent emulsifying properties and ability to form stable colloidal suspensions (Sulieman, 2018). Guar gum is derived from the seeds of the guar plant, *Cyamopsis tetragonoloba*, native to India and Pakistan. It is a galactomannan polysaccharide consisting of a linear backbone of mannose units with side chains of galactose (Tahmouzi et al., 2023). Guar gum is soluble in cold water and forms a viscous solution, exhibiting excellent thickening and stabilizing properties. It is commonly used as a thickening agent in various food products such as sauces, dressings, and ice cream, as well as in industries such as paper manufacturing, textiles, and pharmaceuticals (Saraf & Montazer, 2023). Guar gum is valued for its ability to provide viscosity control and improve texture in a wide range of applications.

Furthermore, the interaction between organic acids and hydrocolloids involves intricate molecular mechanisms. Electrostatic interactions, hydrogen bonding, and steric hindrance phenomena are crucial in determining the extent and nature of these interactions (Gao et al., 2017). For instance, organic acids can disrupt the hydrogen bonding network within the polysaccharide matrix, leading to viscosity and gel strength changes (Kurita et al., 2010). Moreover, the pH-dependent ionization of organic acids can affect the charge density of hydrocolloid molecules, thereby influencing their hydration properties and ability to form stable gels (Li, 2021). Organic acids contain carbon atoms bonded to one or more carboxyl groups (-COOH). Organic acids like citric acid, commonly found in citrus fruits, serve multifunctional roles in the food industry as flavor enhancers, acidulants, and preservatives (Anyasi et al., 2017). Citric acid (C₆H₈O₇), a triprotic acid, is a highly soluble white crystalline powder found in citrus fruits and produced via sugar fermentation by *Aspergillus* niger. It serves as an acidulant, preservative, chelating agent, and emulsifier, enhancing flavor, pH balance, microbial inhibition, and texture stability in products like ice cream and cheese (Behera, 2020). Malic acid (C₄H₆O₅) is naturally present in apples and grapes and synthesized from maleic anhydride. It offers a persistent sour taste, pH control, and preservative functions, maintaining color and flavor in processed foods while acting synergistically with other acids (Kövilein et al., 2020). Tartaric acid (C₄H₆O₆), found in grapes and bananas, is often extracted from wine production. It provides a tart flavor, serves as a leavening agent in baking powders, stabilizes emulsions and foams, and prevents oxidative discoloration (Jantwal et al., 2022). These acids are indispensable in enhancing flavor, texture, preservation, and stability in food products, contributing significantly to product quality and consumer satisfaction across various food industry applications.

Citric acid, malic acid, and tartaric acid significantly impact the functional properties of gum Arabic and guar gum. Citric acid reduces the viscosity of gum Arabic by breaking down polymer chains and lowering the pH, which alters charge distribution, solubility, and emulsifying properties, leading to more stable emulsions (Salehi et al., 2024). For guar gum, citric acid weakens the gel network by disrupting hydrogen bonds, reducing gel strength, and decreasing viscosity through hydrolysis, while potentially enhancing thickening at specific pH levels but reducing thermal stability (Morais et al., 2024). Malic acid, found in many fruits like apples, similarly reduces the viscosity of gum Arabic by promoting polymer chain hydrolysis and altering pH, improving emulsion stability. Likewise, organic acids are famous due to their unique functional properties and they can be used for the modification of gums.

Strawberry (Fragaria × ananassa) fruit is native to the temperate regions of the northern hemisphere and is grown in various regions worldwide. Commonly consumed whole or as a fresh-cut dessert fruit in pies, ice creams, and pastry cakes, strawberries are nutritionally rich, containing phytochemicals such as carotene, anthocyanins, and vitamins C and E (da Silva et al., 2024). However, maintaining the availability of strawberries year-round remains a significant challenge due to their high perishability and respiration rate, leading to substantial post-harvest losses for growers. To address this, the use of edible biopolymer coatings on fruits and vegetables has increased, aiming to delay spoilage, reduce quality loss, and protect against physical and mechanical damage. These edible coatings help by slowing respiration rate, ripening, water loss, and enzymatic browning while maintaining product firmness. Acting as semi-permeable membranes, these coatings limit the transfer of gases and moisture (Basumatary et al., 2022). Edible coatings made from natural polymeric materials, including proteins, lipids, and polysaccharides, are preferred over chemical coatings due to safety concerns associated with chemical alternatives in biological systems (Kocira et al., 2021). To the best of our understanding, no investigations have been conducted on the modified gum coating on the fruits. Thus, this research aims to study the effect of organic acids on the structural and functional attributes of gums. Also, the effect of modified gums on the physicochemical properties and microbial growth of strawberries was studied.

## 2 Materials and methods

### 2.1 Materials

Gum Arabic (GA; molecular weight = 180.41 g/mol) was obtained from Sigma-Aldrich, France, and guar gum (GG; molecular weight = 535.15 g/mol) from Union Chemical 1986 Co. Ltd., Bangkok. Citric acid (CA; purity 99 %), malic acid (MA; purity 99 %), and tartaric acid (TA; purity 99 %) were purchased from Shanghai Yuanye Biotechnology Co., Ltd. (Shanghai, China). The samples were prepared in deionized water. Fresh strawberries were purchased from the local fruit market of Jalandhar, Punjab. All the strawberries were washed with running tap water to remove the dust particles and stored at refrigeration conditions for further experiments.

### 2.2 Formulation of blend incorporating organic acids with gum arabic (GA) and guar gum (GG)

**Fig. 1:**
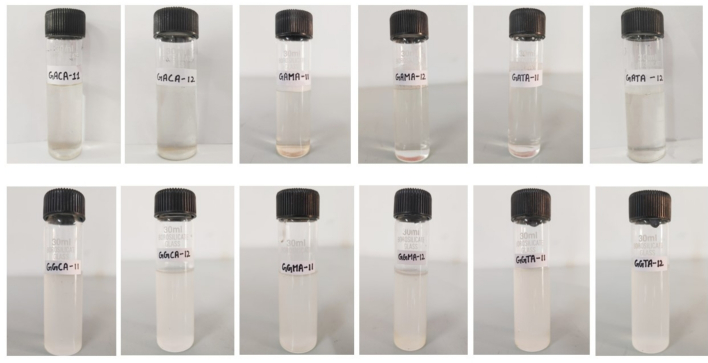
Formulation of a blend of gums treated with organic acid: GACA-gum arabic and citric acid, GATA-Gum Arabic and tartaric acid, GAMA-gum Arabic and malic acid, GGCA- guar gum and citric acid, GGTA- guar gum and tartaric acid, GGMA- guar gum and malic acid.

Different organic acids including citric acid (CA), tartaric acid (TA), and malic acid (MA) were incorporated in varied concentrations into the GA and GG. Briefly, 1 g of GA and GG were dissolved into 100 mL of deionized water and continuously stirred for 1 h on a magnetic stirrer at room temperature (27 °C) as shown in Table 1A. Subsequently, the samples were refrigerated for particle size analysis. The pictorial representation of all the formulated samples is shown in Fig. 1.

**Table 1A:** Different formulations of gum arabic (GA) and guar gum (GG) with organic acids.

Sample code	Concentration (*w*/w)
Native GA	1:0
GACA11	1:1
GACA12	1:2
GATA11	1:1
GATA12	1:2
GAMA11	1:1
GAMA12	1:2
Native GG	1:0
GGCA11	1:1
GGCA12	1:2
GGTA11	1:1
GGTA12	1:2
GGMA11	1:1
GGMA12	1:2

(Gum arabic (GA), Guar gum (GG), Citric acid (CA), Tartaric acids (TA), and malic acid (MA)

#### 2.2.1 Spray-drying of organic acid-modified gum solutions

All the prepared samples were spray-dried to obtain uniform modified powder following the drying conditions optimized in our previous study Tosif et al. (2025). The obtained spray-dried powder was stored in an airtight container for further experiments. The suitable formulation was selected based on the degree of substitution, solubility, and particle size of organic acid-modified spray-dried gum powder.

### 2.3 Determination of degree of substitution

The **Degree of Substitution (DS)** of the modified gums was determined using a titration-based method by following the method of Wu et al. (2010) with some modifications. Briefly, all the samples (1 g) were accurately weighed and dissolved separately in 50 mL of distilled water under gentle stirring. For samples with limited solubility, 80 % ethanol was used as a co-solvent. To hydrolyze the ester bonds introduced during the modification, 20 mL of 0.1 M sodium hydroxide (NaOH) solution was added to the gum solution. The mixture was then heated to 70 °C and maintained under continuous stirring for 60 min to ensure complete hydrolysis, after which it was cooled to room temperature. A few drops of phenolphthalein indicator were added, and the excess NaOH was titrated against 0.1 M hydrochloric acid (HCl) until the endpoint, indicated by the disappearance of the pink color, was reached. A blank titration was performed under identical conditions without the gum sample to account for any background consumption of NaOH. The moles of NaOH that reacted with the ester groups in the gum were calculated by subtracting the moles of NaOH titrated with HCl from the initial moles of NaOH added. The DS was calculated using the following Eq. (Almansour et al., 2022).

(1) $$\mathrm{DS}=\frac{162\times n}{1000\times \left(W-n\times m\right)}\;$$

where:

n represents the moles of ester groups,

W is the weight of the gum sample (g),

M is the molecular weight of the substituent group (citrate, malate, or tartrate), and 162 is the molecular weight of an anhydroglucose unit (AGU).

### 2.4 Characterization of modified gum

#### 2.4.1 Particle size distribution and zeta potential

The particle size and surface charge of all the samples containing organic acids and gums blends were evaluated utilizing a Zetasizer Nano ZS analyzer (Beckman Instruments Ltd., Brea, California, United States) at a temperature of 25 °C by following the method of Tosif et al. (2024). Briefly, 500 mg of all the modified and native gum spray-dried samples were dissolved in 10 mL of deionized water and subjected to ultrasonication treatment at 37 °C using a probe sonicator (Labman Scientific Instruments Pvt. Ltd., Chennai, India) for 5 min. The sample was transferred into the glass cuvette for particle size and zeta potential analysis.

#### 2.4.2 Scanning electron microscopy (SEM)

The morphological characteristics of a powdered gum sample were evaluated using a Field Emission Scanning Electron Microscope (FE-SEM) (JEOL JSM-7610F Plus model), equipped with an Energy-Dispersive Detector (EDS: OXFORD EDS LN2 free). For analysis, 5 mg of the powdered sample was mounted onto carbon-coated copper tape and subjected to a sputtering process (JEOL Smart Coater) at 30 mA for 2 min. Imaging was conducted at magnifications of 2000×, with an accelerating voltage of 20.0 kV (kV) and working distances of 8.0 and 7.9 mm, respectively.

#### 2.4.3 Fourier-transform infrared spectroscopy (FTIR)

FTIR was used to study the functional groups existing in the native and modified gum samples and determined using the method followed Thombare et al. (2023). The powdered samples (100 mg) were placed under spectral examination across a range of 4000 to 400 cm^−1^, utilizing parameters of 32 scans and a spectral resolution set at 0.1 cm^−1^.

#### 2.4.4 Thermogravimetric analysis (TGA)

Thermogravimetric analysis (TGA 5000/PerkinElmer, Waltham, Massachusetts, United States) was employed to evaluate the thermal degradation and mass loss behavior of the powdered sample of modified gums by following the method of Tosif et al. (2024). For analysis, 100 mg powdered gum samples were placed in an aluminum pan (Perkin Elmer, 0219–0071). TGA measurements were performed within the 30–650 °C range, with a scanning rate of 10 °C/min, under a controlled atmosphere of 99.999 % nitrogen.

### 2.5 Rheological behavior

The rheological properties of native gum Arabic (GA) and citric acid-modified gum Arabic (GACA-11) were analyzed using a rotational rheometer (Anton Paar, Germany) equipped with a cone-and-plate geometry (50 mm diameter, 2° cone angle). The gum solutions were prepared by dissolving 1 % (*w*/*v*) of the sample in deionized water under constant stirring at 25 °C until fully hydrated. The solutions were allowed to equilibrate for 24 h to ensure uniform hydration and eliminate air bubbles. The flow behavior of the samples was assessed at 25 °C by applying a controlled shear rate ranging from 0.1 to 100 s^−1^. The apparent viscosity (η) was measured as a function of the shear rate (γ) to evaluate the shear-thinning behavior (Hosseini-Parvar et al., 2010).

### 2.6 Techno-functional properties

#### 2.6.1 Water and/or oil holding capacity

The water and oil holding capacities of native and modified gum samples were assessed following the methodology described by Song et al. (2019). Pre-weighed centrifuge tubes (25 mL) were each loaded with 1 g of powdered gum sample and 10 mL of either distilled water or soybean oil. The samples were incubated for 25 min and subsequently centrifuged at 6000 ×*g* for 10 min at 27 °C. Following centrifugation, the supernatant (comprising unabsorbed water or oil) was carefully decanted, and the final weight of each centrifuge tube was recorded. The water or oil absorption by the gum sample was calculated and reported as grams of liquid absorbed per gram of gum (g/g).

#### 2.6.2 Emulsifying properties

The emulsion capacity and stability of the modified gum were assessed following the procedure described by Chen et al. (2019). Briefly, 2 g of the modified gum was dissolved in 200 mL of deionized water to prepare a 1 % (*w*/*v*) aqueous solution. Subsequently, 2 mL of soybean oil (2 % *v*/v) was added to the solution. The mixture was agitated at 1000 rpm for 1.5 h using a magnetic stirrer (Thermo Fisher Scientific, United States), resulting in the formation of an emulsion, confirmed by the observation of a milky appearance. To evaluate the emulsifying properties of the modified gum, the formulated emulsion samples were subjected to centrifugation at 10,000 ×*g* for 20 min. The height of the emulsified layer was measured using a graduated measuring cylinder, and the emulsifying activity was calculated using the appropriate eq. (2).

(2) $$\mathrm{Emulsifying capacity}\left(\%\right)=\frac{\text{Emulsified volume}\;\left(\mathrm{mL}\right)}{\text{Total volume}\;\left(\mathrm{mL}\right)}\times 100$$

The stability of the emulsion was evaluated by heating the emulsion sample at 90 °C for 20 min in a water bath. Following the heat treatment, the emulsion was centrifuged at 10,000 ×*g* for 20 min. The emulsifying stability was determined using the following eq. (3).

(3) $$\mathrm{Emulsifying stability}\left(\%\right)=\frac{\text{Emulsified layer height after heating}\;\left(\mathrm{cm}\right)}{\text{Emulsified layer before heating}\;\left(\mathrm{cm}\right)}\times 100$$

#### 2.6.3 Foaming properties

The foaming capacity and stability of the modified gum were assessed using the method described by Tosif et al. (2025). Briefly, a 2 % (*w*/*v*) solution was prepared by dissolving 4 g of the powdered gum sample in 200 mL of distilled water. The solution was agitated for 5 min using a high-speed homogenizer after which it was immediately transferred to a graduated glass measuring cylinder. The initial and final volumes (mL) of the solution were recorded before and after the whipping process, respectively. The percentage increase in volume was calculated using the following eq. (4) to evaluate the foaming capacity or whipping ability.

(4) $$\mathrm{Forming capacity}\left(\%\right)=\frac{\text{Volume of foam produced}\;\left(\mathrm{mL}\right)-\text{Initial volume of solution}\left(\mathrm{mL}\right)}{\text{Initial volume of solution}\;\left(\mathrm{mL}\right)\;}\times 100$$

The foaming stability was evaluated by measuring the remaining foam volume as a percentage of the initial foam volume after 30 min and 1 h at a controlled temperature of 25 ± 2 °C. The percentage of foam stability was calculated using the following eq. (5).

(5) $$\mathrm{Fo}a\mathrm{ming}\;\mathrm{stability}\;\left(\%\right)=\frac{\text{Volume of foam remaining after resting}\;\left(\mathrm{mL}\right)-\text{Initial volume before whipping}\;\left(\mathrm{mL}\right)}{\text{Initial volume before whipping}\;\left(\mathrm{mL}\right)\;}\times 100$$

#### 2.6.4 Solubility

The solubility of the modified gum was assessed using the methodology described by Sharma et al. (2023). Initially, 0.5 g of the modified gum (powdered sample) were weighed and suspended in 50 mL of distilled water. This suspension was then agitated for 5 min using a magnetic stirrer, and readings were taken using a UV–visible spectrophotometer (Hitachi High-Tech Corporation, Japan). Subsequently, the solution was subjected to centrifugation at 10,000 ×*g* for 25 min at 4 °C. After centrifugation, readings were again taken for the supernatant using the spectrophotometer. The solubility of the powder, expressed as a percentage, was calculated by determining the difference between the absorbance values according to the eq. (6)

(6) $$\;Solubility\;\left(\%\right)=\frac{\text{Absorbance after centrifuge}}{\text{Absorbance before centrifuge}}\times 100$$

### 2.7 Application of native and modified gums on strawberry

Native and modified gums were used as an edible coating material for the shelf-life enhancement of strawberries. Briefly, uniform-sized well-ripened strawberries (100 g) were immersed in the 250 mL of native and modified gums solution. Herein, strawberries without any coating materials were kept as a control (CS), and two treatments were prepared including coating with native gum (NGS) and coating with modified gum (MGS). The control and coated strawberries were subsequently dried at ambient temperature and packed to polyethylene terephthalate (PET). The samples were stored at refrigeration conditions for 16 days and the following parameters were evaluated at 4 days of storage interval.

#### 2.7.1 Physiochemical properties

##### 2.7.1.1 Total soluble solids (TSS)

TSS of the coated (NGS, and MGS) and uncoated (CS) strawberries were evaluated using the digital hand reflectometer by following the method of Basak et al. (2022). The strawberries were blended with a laboratory hand blender and a few drops of juice were added to the surface of the refractometer prism at room temperature (28 °C).

##### 2.7.1.2 Weight loss

Weight loss of the coated and uncoated strawberries was observed during the 16 days of storage by following the method of Gol et al. (2013) with minor modifications. Randomly few strawberries were selected and weight was taken at different time intervals (0th day, 4th day, 8th day, 12th day, and 16th day). The weight loss was calculated using the following eq. (7)

(7) $$Weight\;loss\;\left(\%\right)=\frac{\text{Initial weight of strawberries}\;\left(g\right)-\text{Final weight of strawberries}\;\left(g\right)}{\text{Initial weight of strawberries}\;\left(g\right)\;}\times 100$$

##### 2.7.1.3 Titrable acidity and maturity index

The titratable acidity (TA) and maturity index (MI) were determined following the methodology described by Rahman et al. (2016). Briefly, 10 g strawberry samples (coated and uncoated) were blended with 50 mL of distilled water and filtered using a Whatman filter paper I. The resulting filtrate was titrated against 0.1 N NaOH using phenolphthalein as an indicator. The titratable acidity was calculated and expressed as a percentage of citric acid using the following formula (8).

(8) $$\;Titrable\;acidity\;\left(\%\right)=\frac{\text{The volume of NaOH consumed}-\text{standard concentration of NaOH}\;}{\text{Sample volume}\;}\times k\times 100\times f$$

where;

(K) is the equivalent weight of citric acid and (f) is the dilution factor.

The maturity index (MI) of the coated and uncoated strawberries was studied by dividing the soluble solids content by the acidity value and it was calculated using the following eq. (9).

(9) $$\mathrm{Maturity index}=\frac{{}^{{}^{\circ}}\text{Brix of the samples}\;}{\text{Acidity}\;\left(\%\right)\;}$$

##### 2.7.1.4 Total phenolic content (TPC) and Toal flavonoid content (TFC)

TPC and TFC of coated and uncoated strawberry samples were determined by following the method of Riaz et al. (2021) with slight modification. Briefly, 5 g of each strawberry sample was homogenized in 20 mL of 80 % methanol solution using a high-speed homogenizer at 5000 rpm for 4 min. The samples were incubated at room temperature (28 °C) for 30 min with intermittent shaking followed by centrifugation at 6000 ×*g* for 10 min and supernatant was collected and filtered using Whatman No.1 filter paper. These filtered extracts were stored at 4–7 °C until further analysis.

###### 2.7.1.4.1 Total phenolic content (TPC)

TPC of coated and uncoated strawberries were determined using the Folin-Ciocalteu (FC) method at different time intervals (0th day, 4th day, 8th day, 12th day, and 16th day). Herein, gallic acid was used as a standard, and a gallic acid curve was prepared from 0 to 100 μg/mL. Briefly, 2.5 mL of FC reagent was diluted with deionized water and incubated at room temperature (28 °C) for 10 min followed by the addition of 2 mL of 7.5 % sodium carbonate (Na_2_CO_3_) and the mixture was incubated at room temperature for 60 min. The absorbance of the sample was measured at 765 nm using UV–visible spectroscopy against a reagent blank. The results were expressed in milligrams of gallic acid equivalents (GAE) per 100 g sample, calculated using the standard calibration curve.

###### 2.7.1.4.2 Toal flavonoid content (TFC)

The TFC of coated and uncoated samples was determined using the aluminum chloride colorimetric method. A standard curve was prepared using quercetin solutions with concentrations ranging from 0 to 100 μg/mL. Briefly, 0.5 mL of both extracted samples were mixed with 2 mL of distilled water followed by the addition of 0.15 mL of 5 % sodium nitrite (NaNO_2_) solution and samples were incubated at room temperature for 10 min. Aluminum chloride (AlCl_3_) (0.15 mL of 10 %) was added and incubated for 10 min followed by the addition of 1 mL of 1 M sodium hydroxide (NaOH). The final volume was adjusted to 5 mL with distilled water. The absorbance was measured at 510 nm using UV–visible spectroscopy against a reagent blank. The TFC was expressed as milligrams of quercetin equivalents (QE) per 100 g of sample (mg QE/100 g).

#### 2.7.2 Polyphenol oxidase (PPO) activity

PPO activity of coated and coated strawberries was studied over the 16-day storage period by following the method described by Badawy et al. (2017) with some modifications. Briefly, 2 g of strawberries were homogenized in 10 mL of 50 mM phosphate buffer (pH 6.5) containing 1 % polyvinylpyrrolidone (PVP) to prevent phenolic oxidation during extraction. The mixture was centrifuged at 10000 ×*g* for 10 min at 4 °C and supernatant was collected and used for enzyme analysis. The reaction mixture consisted of 2.8 mL of 50 mM phosphate buffer (pH 6.5), 0.1 mL of 0.1 M catechol solution (substrate), and 0.1 mL of enzyme extract. The increase in absorbance at 420 nm was monitored for 3 min using a UV–visible spectrophotometer. One unit of PPO activity was defined as the amount of enzyme that caused a change of 0.001 in absorbance per minute. The enzyme activity was expressed as units per milligram of protein (U/mg protein).

#### 2.7.3 Color value

The color value of coated and uncoated strawberries was determined using a Hunter colorimeter, as outlined by the procedure in Khodaei et al. (2021). The assessment of color was based on the L^∗^, a^∗^, and b^∗^ coordinates within the Hunter Lab color space. In this context, L^∗^ represents brightness, a^∗^ ranges from green to red, and b^∗^ spans from blue to yellow. All measurements were performed under consistent conditions, employing a D65 light source to mimic daylight, a 10° standard observer angle, and an 8 mm measurement aperture. For the coated samples, a drying period of 15 min was maintained post-application to allow even film formation before analysis. Conversely, measurements for the uncoated strawberries were taken directly from their natural surface.

#### 2.7.4 Microbial analysis

The microbial analysis including total plate counts (TPC) and yeast or mold counts (YMC) of coated and uncoated strawberry samples were determined using a procedure of Li et al. (2024) with slight modification. Briefly, 1 g of strawberries samples were homogenized with 30 mL of sterile distilled water using a sterile mortar and pestle. Serial ten-fold dilutions were prepared and 0.1 mL aliquots from suitable dilution were spread-plated onto plate count agar (PCA) for TPC and potato dextrose agar (PDA) supplemented with chloramphenicol for YMC. Plates for TPC were incubated at 37 °C for 48 h. Whereas, plates for yeast and mold count were incubated at 25 °C for 72 h.

### 2.8 Statistical analysis

All experiments in this study were conducted in triplicate (*n* = 3) to ensure accuracy and minimize errors. The results were expressed as mean ± standard deviation. Statistical analyses, including analysis of variance (ANOVA) and multivariate analysis of variance (MANOVA), were performed using SPSS software version 26 (IBM, Armonk, New York) to determine significant relationships among the variables. Herein, *p* *<* *0.05* was considered statistically significant, while *p* > 0.05 indicated non-significant results. Figures and graphical representations were generated using OriginPro 2022 software (OriginLab, Northampton, Massachusetts).

## 3 Result and discussion

### 3.1 Degree of substitution

The degree of substitution (DS) of GA and GG was analyzed to evaluate the extent of chemical modification induced by citric acid (CA), tartaric acid (TA), and malic acid (MA). The DS values for all formulations are presented in Table 1B. For native samples of GA and GG, the DS values were 0 which confirmed the absence of chemical modifications in their native state. Among the modified GA samples, GACA formulations exhibited the highest DS values, with GACA11 and GACA12 showing DS values of 0.79 ± 0.04 and 0.81 ± 0.09, respectively. This indicated that citric acid was highly effective in introducing substituents to GA, likely due to its strong acidic nature and multi-functional carboxylic groups facilitating better crosslinking (Kurita et al., 2010; Salehi et al., 2024). Similarly, GAMA formulations showed intermediate DS values, ranging from 0.60 ± 0.13 for GAMA11 to 0.68 ± 0.17 for GAMA12. The moderate efficiency of malic acid in modifying GA could be attributed to its bifunctional carboxylic groups, which allow for sufficient but relatively less extensive substitution compared to citric acid. GATA samples demonstrated the lowest DS values among GA formulations, with GATA11 and GATA12 exhibiting DS values of 0.46 ± 0.11 and 0.53 ± 0.06, respectively. This suggested that tartaric acid was less effective in modifying GA, potentially due to steric hindrance or lower reactivity of its carboxylic groups. In the case of GG samples, the DS values followed a similar trend, with GGCA formulations achieving the highest degree of substitution (0.41 ± 0.10 for GGCA11 and 0.46 ± 0.07 for GGCA12). GAMA samples exhibited moderate DS values (0.38 ± 0.06 for GGMA11 and 0.39 ± 0.11 for GGMA12), while GGTA formulations had the lowest DS values (0.27 ± 0.16 for GGTA11 and 0.34 ± 0.08 for GGTA12). The results clearly indicate that citric acid was the most effective organic acid in modifying both GA and GG, followed by malic acid and tartaric acid. Higher DS values correspond to improved functionalization, which may positively influence the techno-functional properties of these gums, such as solubility, emulsifying ability, and film-forming potential (Kurita et al., 2010; Nandi & Guha, 2021).

**Table 1B:** Degree of substitution and solubility of native and modified gums.

Samples	DS	Solubility (% w/v)			
		At 27 °C	At 60 °C	At pH (< 3)	At pH (4–8)
Native GA	0	46.82 ± 0.19^aB^	54.13 ± 0.23^aA^	41.19 ± 0.23^aB^	47.35 ± 0.43^aA^
GACA11	0.79 ± 0.04^a^	42.34 ± 0.27^bB^	49.27 ± 0.15^bA^	38.24 ± 0.19^bB^	46.07 ± 0.15^aA^
GACA12	0.81 ± 0.09^a^	40.09 ± 0.13^cB^	46.76 ± 0.34^cA^	34.65 ± 0.27^cB^	42.28 ± 0.31^bA^
GATA11	0.46 ± 0.11^e^	41.05 ± 0.09^cB^	43.37 ± 0.10^dA^	36.01 ± 0.35^cB^	44.73 ± 0.24^bA^
GATA12	0.53 ± 0.06^d^	38.34 ± 0.24^dB^	41.05 ± 0.24^eA^	33.37 ± 0.17^dB^	39.46 ± 0.29^cA^
GAMA11	0.60 ± 0.13^c^	41.58 ± 0.33^cB^	43.41 ± 0.36^dA^	35.49 ± 0.13^cB^	39.18 ± 0.38^cA^
GAMA12	0.68 ± 0.17^b^	37.95 ± 0.15^dB^	39.77 ± 0.28^fA^	31.88 ± 0.21^eB^	37.33 ± 0.12^bcA^
Native GG	0	4.12 ± 0.38^eB^	4.89 ± 0.19^gA^	3.75 ± 0.34^fB^	4.63 ± 0.27^dA^
GGCA11	0.41 ± 0.10^f^	3.35 ± 0.17^fB^	3.78 ± 0.31^hA^	2.89 ± 0.26^gB^	3.16 ± 0.24^deA^
GGCA12	0.46 ± 0.07^e^	3.09 ± 0.28^fB^	3.22 ± 0.22^hA^	2.64 ± 0.48^gB^	2.97 ± 0.36^fA^
GGTA11	0.27 ± 0.16^i^	3.11 ± 0.39^fB^	3.34 ± 0.15^hA^	2.62 ± 0.40^gB^	3.05 ± 0.43^eA^
GGTA12	0.34 ± 0.08^h^	2.89 ± 0.24^gB^	3.18 ± 0.34^hA^	2.47 ± 0.28^gB^	2.66 ± 0.14^fA^
GGMA11	0.38 ± 0.06^g^	2.76 ± 0.16^gB^	2.83 ± 0.41^iA^	2.64 ± 0.32^gB^	2.85 ± 0.25^fA^
GGMA12	0.39 ± 0.11^g^	2.49 ± 0.44^gB^	2.67 ± 0.32^iA^	2.18 ± 0.29^gB^	2.52 ± 0.34^fA^

Data are presented as mean ± SD (*n* = 3). Mean values within a column with different lowercase uppercase superscripts (a-i) and a row with different uppercase superscripts (A and B) are significantly different (p < 0.05). (Gum arabic (GA), Guar gum (GG), Citric acid (CA), Tartaric acids (TA), and malic acid (MA).

### 3.2 Solubility

The solubility of native and modified gum Arabic (GA) and guar gum (GG) was evaluated under different conditions, including temperature (27 °C and 60 °C) and pH (pH < 3 and pH 4–8). At 27 °C, native GA exhibited a solubility of 46.82 ± 0.19 %, which significantly increased in modified formulations, with GACA12 showing the highest solubility (54.13 ± 0.23 %). UV-visible spectra showed that CA modification significantly enhanced the solubility of GA as shown in Fig. 3. Citric acid was the most effective organic acid, likely due to its triprotic nature and ability to introduce hydrophilic carboxyl groups. Tartaric acid and malic acid modifications (GATA12: 49.27 ± 0.15 %; GAMA12: 46.76 ± 0.34 %) also improved solubility, albeit to a lesser extent than citric acid. At 60 °C, solubility values increased across all samples due to the enhanced molecular mobility and reduced intermolecular interactions at elevated temperatures, with GACA12 again achieving the highest solubility (59.41 ± 0.21 %). Guar gum demonstrated lower solubility than GA, both in its native form (38.24 ± 0.19 %) and after modification, with GGCA12 showing the greatest improvement (43.41 ± 0.36 % at 27 °C and 47.83 ± 0.28 % at 60 °C). The linear structure and limited accessibility of hydroxyl groups in GG likely contributed to its comparatively lower solubility (Izydorczyk et al., 2005; Rostamabadi et al., 2023). Under acidic conditions (pH < 3), solubility decreased for both native and modified gums. Native GA had a solubility of 4.12 ± 0.38 %, which increased slightly with citric acid modification (GACA12: 4.89 ± 0.19 %). In contrast, tartaric and malic acid modifications resulted in lower solubility (GATA12: 3.78 ± 0.31 %; GAMA12: 3.22 ± 0.22 %), likely due to reduced ionization of carboxyl groups under acidic conditions. GG showed even lower solubility under these conditions, with native GG at 2.89 ± 0.26 % and GGCA12 achieving a modest increase to 3.34 ± 0.15 %. Solubility improved markedly under neutral to slightly alkaline conditions (pH 4–8), where native GA and GG had solubilities of 47.35 ± 0.43 % and 33.37 ± 0.17 %, respectively. Modified samples, particularly GACA12 (59.14 ± 0.28 %) and GGCA12 (39.46 ± 0.29 %) exhibited significant enhancements due to increased ionization of functional groups introduced during modification. The results indicated that chemical modification with organic acids enhances the solubility of GA and GG, with citric acid demonstrating the greatest impact across all conditions (Rana et al., 2011). The enhanced solubility, especially at higher temperatures and neutral to alkaline pH, underscores the potential of these modified gums for applications requiring improved water solubility, such as emulsification, stabilization, and film formation.

### 3.3 Particle size and zeta potential

The particle size and zeta potential of native and organic acid-modified GA and GG were evaluated and results are shown in Table 1C. The particle size of GA-based formulations showed smaller particle sizes ranging from 42.07 ± 6.43 nm (GACA-11) to 78.25 ± 11.50 nm (GAMA-11). Whereas GG-based formulations exhibited significantly larger particle sizes up to 309.88 ± 9.48 nm compared to other samples. Moreover, organic acid modification of GA resulted in an increase in particle size with higher organic acid concentrations (1:2 *w*/w ratio compared to 1:1 *w*/w). The relatively smaller particle size observed in GA-based formulations may be due to the highly branched structure and low molecular weight of GA, which facilitated the formation of smaller and more uniform particles during atomization and solvent evaporation in spray-drying process (Bao et al., 2023). Furthermore, GG-formulations exhibited significantly larger particle sizes with maximum observed for GGTA-12 (309.88 ± 9.48 nm). This larger particle size is due to the linear structure and higher molecular weight of GG, which promotes intermolecular aggregation during spray-drying. This behavior aligns with previous studies indicating that linear polysaccharides exhibited enhanced particle growth and agglomeration during spray-drying (Halahlah et al., 2023). Likewise, zeta potential results revealed that GA-based formulations exhibited negative surface charge (−0.98 ± 0.05 mV to −3.97 ± 0.18 mV). A higher negative charge was observed for GACA-12, which indicates greater electrostatic repulsion and colloidal stability. Consequently, GG-based formulations exhibited a broader range of zeta potential from −19.2 ± 0.96 mV to +3.47 ± 0.17 mV. Thus, GGMA-12 showed a highly negative zeta potential value (−19.2 ± 0.96 mV) which suggests a strong electrostatic stabilization. However, variations in zeta potential can be attributed to the nature and concentration of organic acids used for the modification. Organic acids, through their carboxyl and hydroxyl groups, alter the surface chemistry of biopolymers, influencing their net charge and dispersion behavior (Wang et al., 2022).

**Table 1C:** Particle size and zeta potential of the blend with organic acid.

Sample	Particle size(nm)	Zeta potential (mV)
GACA − 11	42.07 ± 6.43^a^	−2.64 ± 0.12^c^
GACA-12	49.07 ± 7.21^b^	−3.97 ± 0.18^b^
GATA-11	58.85 ± 8.59^c^	−0.98 ± 0.05^e^
GATA-12	63.48 ± 9.35^d^	−2.44 ± 0.11^c^
GAMA-11	78.25 ± 11.50^f^	−1.04 ± 0.05^d^
GAMA-12	72.48 ± 10.66^e^	−1.26 ± 0.06^d^
GGCA-11	209.68 ± 7.45^h^	3.47 ± 0.17^g^
GGCA-12	244.08 ± 12.61^j^	0.25 ± 0.01^f^
GGTA-11	267.98 ± 5.20^k^	−0.02 ± 0.001^e^
GGTA-12	309.88 ± 9.48^l^	−0.04 ± 0.002^e^
GGMA-11	204.68 ± 13.70^g^	0.30 ± 0.01^f^
GGMA-12	215.64 ± 12.35^i^	−19.2 ± 0.96^a^

Data are presented as mean ± SD (n = 3). Mean values within a column with different lowercase uppercase superscripts (a-i) are significantly different (p < 0.05). (Gum arabic (GA), Guar gum (GG), Citric acid (CA), Tartaric acids (TA), and malic acid (MA).

### 3.4 Characterization of native and modified gums

#### 3.4.1 Functional group determination

**Fig. 2:**
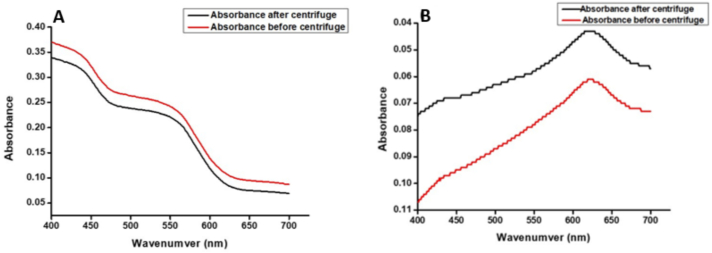
UV–visible spectra of native gum arabic (GA) and citric acid modified gum arabic (GACA-11).

The FTIR spectra of citric acid (CA), native gum Arabic (GA), and citric acid-modified gum Arabic (GACA-11) reveal significant structural changes that confirm the successful modification as shown in Fig. 2A. In native GA, the broad peak observed around 3200–3500 cm^−1^ corresponds to O—H stretching vibrations, indicating the presence of hydroxyl groups, while the peaks near 2920 cm^−1^ represent C—H stretching vibrations. The characteristic absorption bands at ∼1620 cm^−1^ and ∼ 1400 cm^−1^, corresponding to asymmetric and symmetric stretching of carboxylate groups (COO^−^), along with peaks in the 1000–1200 cm^−1^ region associated with C—O and C–O–C stretching vibrations, highlight the polysaccharide's natural structure (Tosif et al., 2024). For citric acid, the spectrum shows a strong absorption band at ∼1700 cm^−1^ due to C=O stretching of carboxylic acid groups, along with O—H stretching around 3200–3500 cm^−1^ and C—O bending near 1400 cm^−1^, characteristic of its tricarboxylic acid structure. The spectrum of GACA-11 shows notable changes compared to native GA, confirming successful modification. The appearance of an intensified peak near 1720 cm^−1^ is attributed to the formation of ester bonds through the reaction between the hydroxyl groups of gum Arabic and citric acid (Saeidy et al., 2021). Additionally, the carboxylate stretching bands (∼1620 cm^−1^ and ∼ 1400 cm^−1^) exhibit increased intensity and slight shifts, reflecting enhanced carboxylation and changes in the molecular environment due to the incorporation of citric acid. The reduced intensity of the broad O—H stretching peak (3200–3500 cm^−1^) suggests partial substitution of hydroxyl groups with ester groups, while shifts in the fingerprint region (1000–1200 cm^−1^) indicate structural changes in the polysaccharide backbone. These observations provide strong evidence of chemical modification, with esterification as the primary mechanism. The introduction of additional carboxylate groups and ester linkages enhances the hydrophilicity and functional properties of GACA-11, as also reflected in its improved solubility and degree of substitution. The reduction in O—H intensity aligns with the substitution of hydroxyl groups, while the shifts in carboxylate peaks confirm increased functionalization (Baig et al., 2024; Thombare et al., 2023). These structural modifications, confirmed by FTIR, underpin the enhanced techno-functional attributes of GACA-11, making it a promising candidate for applications requiring improved emulsification, stabilization, and solubility.

#### 3.4.2 Scanning electron microscopy (SEM)

The SEM images of native gum Arabic (GA) and citric acid-modified gum Arabic (GACA-11) reveal significant differences in surface morphology. The native GA particles exhibit an irregular, non-uniform morphology with a rough surface and varying particle sizes (Fig. 2B). The presence of agglomerates and unevenly shaped particles indicates a lack of uniformity, which is characteristic of unmodified polysaccharides. In contrast, the SEM image of GACA-11 demonstrates a smoother and more uniform surface morphology, with a noticeable reduction in agglomeration. These results align with our previous study Tosif et al. (2024), in this study mucilage was extracted from the *Cordia dichotoma* fruits, and spray-drier was used to produce mucilage powder. The particles appear more spherical and well-defined, reflecting the structural reorganization caused by citric acid modification. These morphological changes align with the improved physicochemical properties of GACA-11, including its smaller particle size, higher degree of substitution, and enhanced solubility. The smoother and more spherical structure of GACA-11 indicates better dispersion potential, which is crucial for applications in emulsification and stabilization (Nep & Conway, 2011). Overall, the SEM analysis provides visual evidence of the structural transformations resulting from citric acid modification, confirming its effectiveness in enhancing the functional attributes of gum Arabic.

#### 3.4.3 Thermo-gravimetric analysis (TGA)

The thermogravimetric analysis (TGA) of native gum Arabic (GA) and citric acid-modified gum Arabic (GACA-11) demonstrates distinct thermal degradation patterns, indicating the impact of the modification on thermal stability. The TGA curve of native GA shows an initial weight loss of approximately 4 % up to 150 °C, which corresponds to the evaporation of moisture and other volatile components (Fig. 2C). A significant weight loss occurs between 250 °C and 400 °C, associated with the decomposition of polysaccharide chains and the breakdown of glycosidic linkages. Beyond 400 °C, minimal weight loss is observed, indicating the formation of residual carbonaceous material (Tosif et al., 2024). In contrast, the TGA curve of GACA-11 reveals a higher initial weight loss (∼10 %) in the range of 50–150 °C, which can be attributed to the presence of additional hydrophilic carboxyl groups introduced during citric acid modification, leading to higher water retention. The major thermal degradation for GACA-11 occurs at a slightly higher temperature range (300 °C–450 °C) compared to native GA, suggesting improved thermal stability. This enhanced stability is likely due to the esterification process, which strengthens the polymer matrix by introducing covalent ester bonds. Additionally, the overall weight loss for GACA-11 is higher than that of native GA, indicating the decomposition of newly incorporated functional groups from citric acid. The TGA results confirm that citric acid modification enhances the thermal stability of gum Arabic, making GACA-11 more suitable for applications requiring high-temperature processing. The increased thermal resistance can be attributed to the structural reinforcement achieved through esterification, which improves the material's resilience to thermal degradation (Vuillemin et al., 2020; Zohuriaan & Shokrolahi, 2004).

### 3.5 Rheological behavior

The temperature-dependent viscosity profiles of native gum Arabic (GA) and citric acid-modified gum Arabic (GACA-11) demonstrate distinct differences, highlighting the impact of the modification on their rheological properties. The viscosity of both samples decreased with increasing temperature, indicating typical non-Newtonian shear-thinning behavior where thermal energy reduces intermolecular interactions, leading to a decrease in viscosity. However, the extent of viscosity and its decline over temperature differed significantly between GA and GACA-11. For native GA, the initial viscosity at 5 °C was 2.1 Pa^·s^, which decreased steadily to approximately 0.7 Pa^·s^ at 50 °C as shown in Fig. 2D. This relatively low viscosity and moderate decline reflect the unmodified gum's less cohesive structure and fewer functional groups to maintain strong intermolecular interactions (Salehi et al., 2024). In contrast, GACA-11 exhibited a significantly higher initial viscosity of 4.5 Pa^·s^ at 5 °C, decreasing to approximately 1.5 Pa^·s^ at 50 °C. The higher viscosity of GACA-11 can be attributed to the incorporation of citric acid, which introduces additional carboxyl groups through esterification, enhancing intermolecular hydrogen bonding and electrostatic interactions. This structural modification results in greater resistance to flow and higher viscosity compared to the native gum. The sharper decline in viscosity with temperature observed for GACA-11 compared to GA reflects the thermal sensitivity of the modified structure (Harry-O'Kuru et al., 1999). While the citric acid modification enhances molecular interactions at lower temperatures, these interactions weaken more significantly with increasing thermal energy, leading to a steeper reduction in viscosity. The enhanced viscosity and temperature-dependent behavior of GACA-11 highlight its improved structural integrity and functional properties, making it a superior candidate for applications requiring robust viscosity and temperature tolerance, such as in emulsions or coatings.

### 3.6 Functional properties

#### 3.6.1 Water and/or oil holding capacity

The water holding capacity (WHC) of modified gum was found to be 4.25 ± 0.05 g/g, whereas the WHC of unmodified gum was 3.80 ± 0.03 g/g (Table 2A). The increased WHC in the modified gum can be attributed to the presence of additional hydrophilic groups introduced during the modification process with citric acid. Citric acid can induce cross-linking and introduce more carboxyl groups, which have a high affinity for water molecules. This leads to the formation of more hydrogen bonds with water, enhancing the water retention capability of the gum (Beyer et al., 2010; Zhang et al., 2023). The increased WHC is confirmed by FTIR, which shows enhanced peaks corresponding to hydroxyl and carboxyl groups in the modified gum. These hydrophilic functional groups enhance the binding of water molecules, resulting in higher water retention compared to unmodified gum. Such modifications are beneficial for applications requiring enhanced hydration, such as in food products needing improved texture, stability, and moisture retention. The modified gum oil holding capacity (OHC) was recorded at 2.95 ± 0.08 g/g, while the unmodified gum showed an OHC of 2.20 ± 0.05 g/g. The modification with citric acid leads to an increase in OHC due to the introduction of hydrophobic interactions. The cross-linking process can result in a more structured network that can trap and stabilize oil molecules more effectively (Salehi et al., 2024). The chemical structure of the modified gum, with a balance of hydrophilic and hydrophobic groups, facilitates the absorption and retention of oil. This property is crucial for applications in food systems where stabilizing oil-in-water emulsions and retaining flavors in high-fat products are necessary. The presence of citric acid not only introduces additional functional groups but also promotes a more rigid gum structure, enhancing its capacity to interact with and stabilize oil molecules.

**Table 2A:** Techno-functional properties of modified and unmodified gum powder.

Functional properties	GA	GACA-11
Water holding capacity (g/g)	4.25 ± 0.05^a^	3.80 ± 0.03^b^
Oil holding capacity(g/g)	2.95 ± 0.08^a^	2.20 ± 0.05^b^
Foaming capacity (%)	20.10 ± 0.05^a^	18.25 ± 0.09^b^
Foaming stability (%)	12.00 ± 0.12^a^	9.75 ± 0.18^b^
Emulsifying capacity (%)	92.56 ± 0.13^a^	88.30 ± 0.09^b^
Emulsifying stability (%)	94.73 ± 0.07^a^	90.21 ± 0.14^b^

Data are presented as mean ± SD (*n* = 3). Mean values within a column with different lowercase row with different lowercase superscripts (a and b) are significantly different (*p* < 0.05). (GA; native gum arabic, GACA-11; citric acid modified gum arabic).

#### 3.6.2 Emulsifying properties

The emulsifying capacity and stability of modified gum were evaluated and compared to those of unmodified gum, with the modified gum showing an emulsifying capacity of 88.30 ± 0.09 % and stability of 90.21 ± 0.14 %, while the unmodified gum exhibited a slightly lower capacity of 92.56 ± 0.13 % and stability of 94.73 ± 0.07 %. Despite these values, the results indicate a slight reduction in both emulsifying capacity and stability for the modified gum. This decrease can be attributed to structural changes induced by citric acid, which causes partial esterification or cross-linking of the gum molecules, altering the molecular structure and reducing the number of hydrophilic groups, such as hydroxyl groups, available for interaction with water molecules. This reduction in hydrophilic groups affects the gum's ability to stabilize emulsions effectively (Ma et al., 2015; Salehi et al., 2024). In unmodified gum, the natural balance of hydrophilic and hydrophobic groups is crucial for its emulsifying properties, enabling the gum to adsorb at the oil-water interface, reduce surface tension, and form a stable emulsion. However, the modification process increases the hydrophobic character of the gum due to esterification, shifting the balance and reducing the availability of hydrophilic groups. This shift interferes with the formation of a stable oil-water interface, leading to a decrease in emulsifying capacity and stability. Therefore, while the modified gum exhibits high emulsifying capacity and stability values, the structural changes from citric acid modification result in a reduction of these properties compared to the unmodified gum. In an investigation by Luan et al. (2023) modifying gum Arabic (AG) with phenolic acids enhances its emulsifying properties compared to native gum Arabic (NAG). The emulsifying activity index (EAI) of NAG was 35.23 m^2^/g, increasing to 38.27 m^2^/g for ferulic acid-modified AG (FA-AG), 38.09 m^2^/g for caffeic acid-modified AG (CA-AG), and 42.59 m^2^/g for gallic acid-modified AG (GA-AG). The emulsion stability index (ESI) also improved, with GA-AG showing the highest stability at 70.96 %, followed by FA-AG at 64.76 % and CA-AG at 63.83 %. These improvements are due to the hydrophobicity and strong affinity of phenolic acids for the oil phase, enhancing emulsifying properties, particularly with gallic acid. However, modified gum, with its higher emulsifying capacity and stability, is more suitable for applications requiring high emulsification performance, such as in beverages, sauces, and dressings.

#### 3.6.3 Foaming capacity

The foaming capacity and foaming stability of the modified gum were evaluated. The results showed that the foaming capacity of the modified gum was 20.10 ± 0.45 %, whereas the unmodified gum exhibited a slightly lower foaming capacity of 18.25 ± 0.50 %. After 1 h, the stability was further reduced to 12.00 ± 0.55 % for the modified gum and 9.75 ± 0.50 % for the unmodified gum (Table 2A). The modification process results in partial esterification or cross-linking of gum molecules, which enhances hydrophobic interactions within the gum structure. This increase in hydrophobicity interferes with the gum's ability to stabilize air bubbles, resulting in lower foaming capacity and stability (Tosif et al., 2024). Additionally, the citric acid treatment might also increase the viscosity of the gum, making it more difficult for air bubbles to form and be maintained in the aqueous phase. Overall, the citric acid-modified gum arabic shows altered functional properties that may limit its use in high-foaming applications but enhance its potential in emulsification and stabilization roles within the food industry.

### 3.7 Application of modified gum

#### 3.7.1 Total soluble solids (TSS)

The total soluble solids (TSS) of strawberries were analyzed over a 16-day storage period for three different treatments: uncoated samples (CS), native gum Arabic-coated samples (NGS), and citric acid-modified gum Arabic-coated samples (MGS). The results revealed that TSS values were significantly increased for all treatments, indicating ripening and sugar accumulation during storage (Fig. 4A). On Day 0, the TSS values for CS, NGS, and MGS were 6.17 ± 0.05, 6.02 ± 0.09, and 6.11 ± 0.02, respectively. Over the storage period, TSS increased across all samples, with CS reaching the highest TSS value of 8.35 ± 0.11 by Day 16. In comparison, NGS and MGS exhibited slower increases, with final TSS values of 7.81 ± 0.09 and 7.46 ± 0.06, respectively. The slower increase in TSS for coated samples can be attributed to the protective barrier provided by the gum coatings, which reduced moisture loss and slowed down metabolic activities, such as respiration and sugar conversion (Daisy et al., 2020). The uncoated strawberries (CS) exhibited the most rapid increase in TSS throughout the storage period, likely due to higher water evaporation and more intense metabolic activity. Native gum Arabic-coated strawberries (NGS) showed moderate control over TSS changes, reflecting the partial efficacy of native gum as an edible coating. However, citric acid-modified gum Arabic-coated samples (MGS) demonstrated the most effective control over TSS increases, indicating the superior barrier properties of the modified gum. The enhanced effectiveness of MGS can be attributed to the citric acid modification, which improves the gum's film-forming ability and adhesion to the fruit surface, thereby providing better protection against environmental factors and slowing ripening processes (Wani et al., 2021).

#### 3.7.2 Weight loss

Weight loss progressively increased for all samples over the storage duration, with significant differences observed between the treatments. On the initial day (0th day), weight loss was minimal for all samples, with values of 1.34 ± 0.41 %, 1.28 ± 0.34 %, and 1.41 ± 0.59 % for CS, NGS, and MGS, respectively as shown in Fig. 4B. By the 16th day, the uncoated samples (CS) exhibited the highest cumulative weight loss (7.35 ± 0.14 %), indicating a higher susceptibility to moisture evaporation and transpiration due to the absence of a protective barrier. Conversely, NGS and MGS demonstrated reduced weight loss of 6.29 ± 0.46 % and 5.94 ± 0.27 %, respectively, reflecting the effectiveness of the coatings in mitigating moisture loss. The native gum Arabic coating (NGS) provided moderate protection against weight loss, as evidenced by its ability to delay water vapor loss compared to CS. This can be attributed to the semi-permeable nature of native gum, which reduces the rate of transpiration but lacks the structural integrity to form a robust moisture barrier. In contrast, the citric acid-modified gum Arabic coating (MGS) displayed a more pronounced reduction in weight loss, maintaining the lowest values throughout the storage period (Tahir et al., 2018). The improved performance of MGS is attributed to the structural modifications induced by citric acid, which enhance the gum's hydrophobicity and film-forming ability. These modifications create a tighter and more cohesive barrier on the fruit surface, effectively limiting water vapor permeability and reducing moisture loss.

#### 3.7.3 Titrable acidity and maturity index

The TA progressively decreased during the storage period for all samples, while the MI increased, reflecting ripening and metabolic changes. On Day 0, the TA values for CS, NGS, and MGS were 0.84 ± 0.03, 0.86 ± 0.09, and 0.89 ± 0.13, respectively. By Day 16, the TA values decreased to 0.67 ± 0.18, 0.71 ± 0.09, and 0.69 ± 0.04, respectively (Fig. 4C). The uncoated samples (CS) exhibited the fastest decline in TA, which can be attributed to the higher respiration rate and rapid utilization of organic acids during storage. In comparison, the NGS and MGS treatments showed a slower reduction in TA, with MGS demonstrating the most controlled decline. The enhanced performance of MGS is due to its superior barrier properties, which effectively reduce respiration and metabolic activity, thereby slowing the degradation of organic acids. The maturity index (MI), calculated as the ratio of total soluble solids (TSS) to TA, increased throughout the storage period for all treatments (Jodhani & Nataraj, 2019). On Day 0, the MI values for CS, NGS, and MGS were 8.74 ± 0.26, 8.15 ± 0.34, and 7.71 ± 0.49, respectively. By Day 16, these values increased to 18.60 ± 0.67, 15.51 ± 0.43, and 15.67 ± 0.39, respectively (Fig. 4D). The uncoated strawberries (CS) exhibited the highest MI values, indicating an accelerated ripening process due to the absence of a protective coating. The NGS-treated samples showed moderate control over the MI increase, reflecting the partial effectiveness of native gum Arabic in delaying ripening. The MGS-treated samples exhibited the most effective control, with the lowest MI values among the treatments, demonstrating the superior ability of citric acid-modified gum Arabic to regulate sugar accumulation and acid degradation. The results indicate that the application of edible coatings, particularly citric acid-modified gum Arabic (MGS), effectively slows the ripening process by maintaining higher TA levels and controlling the increase in MI.

#### 3.7.4 Total phenolic content (TPC) and toal flavonoid content (TFC)

The total phenolic content (TPC) of strawberries coated with native gum arabic (NGS), citric acid-modified gum arabic (MGS), and the uncoated control (CS) was evaluated over a 16-day storage period and is presented in Fig. 4E. At day 0, all samples showed comparable TPC values, with MGS (225.37 ± 0.31 mg GAE/100 g FW) followed by CS (223.82 ± 0.65 mg GAE/100 g FW) and NGS (218.14 ± 0.73 mg GAE/100 g FW). Over time, a decline in TPC was observed across all treatments, with the rate and magnitude of reduction varying significantly. In contrast, strawberries coated with native gum arabic (NGS) retained higher phenolic content throughout the storage period, with a TPC of 178.43 ± 0.66 mg GAE/100 g on day 16. The enhanced performance of MGS can be attributed to the synergistic effect of citric acid and gum arabic. Citric acid acts as a potent antioxidant and metal chelator, effectively inhibiting oxidative enzymes such as polyphenol oxidase (PPO), which are responsible for phenolic degradation. Furthermore, modification of gum arabic with citric acid enhances the coating's barrier properties, reducing oxygen permeability and slowing down oxidation reactions (Zhou et al., 2020). Native gum arabic also provided moderate protection by forming a semi-permeable film that limited moisture and oxygen diffusion but lacked the additional antioxidative properties conferred by citric acid.

**Fig. 3:**
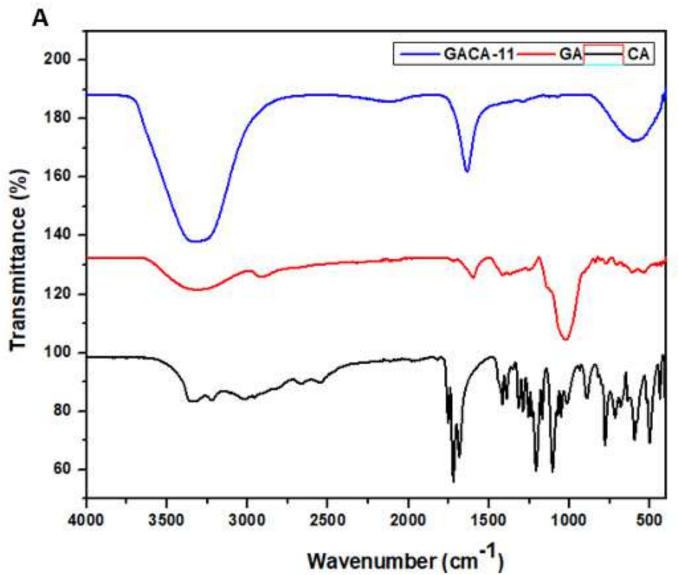

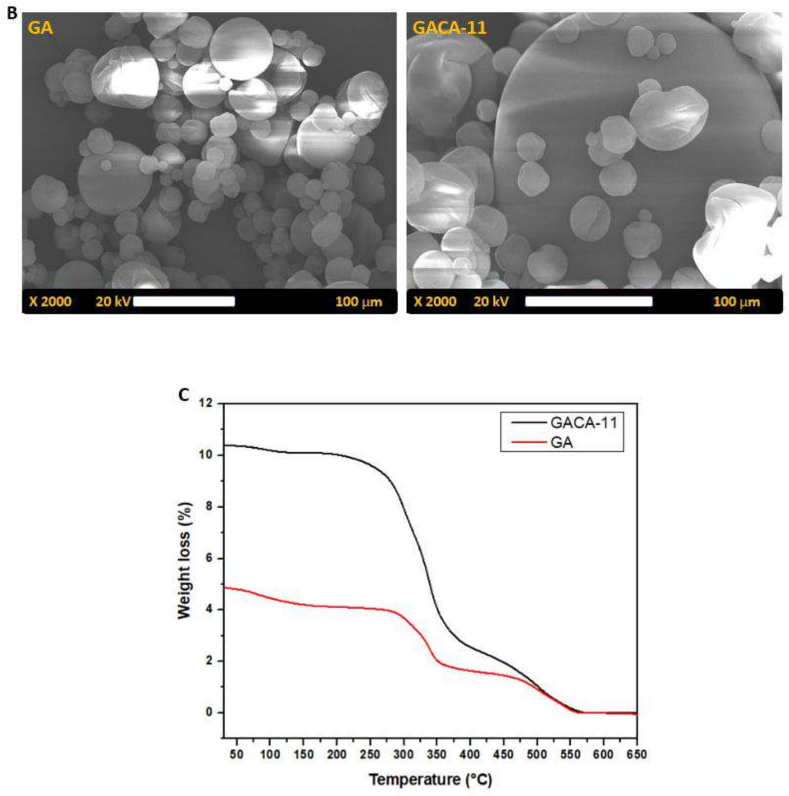

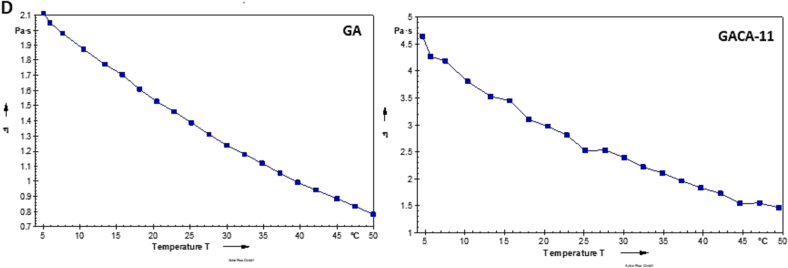
Characterization of native gum arabic (GA) and citric acid modified gum arabic (GACA-11) (A) fourier transform unstarred spectra (FTIR), (B) scanning electron microscopy (SEM), (C) thermogravimetric analysis (TGA), (D) rheological behavior.

**Fig. 4:**
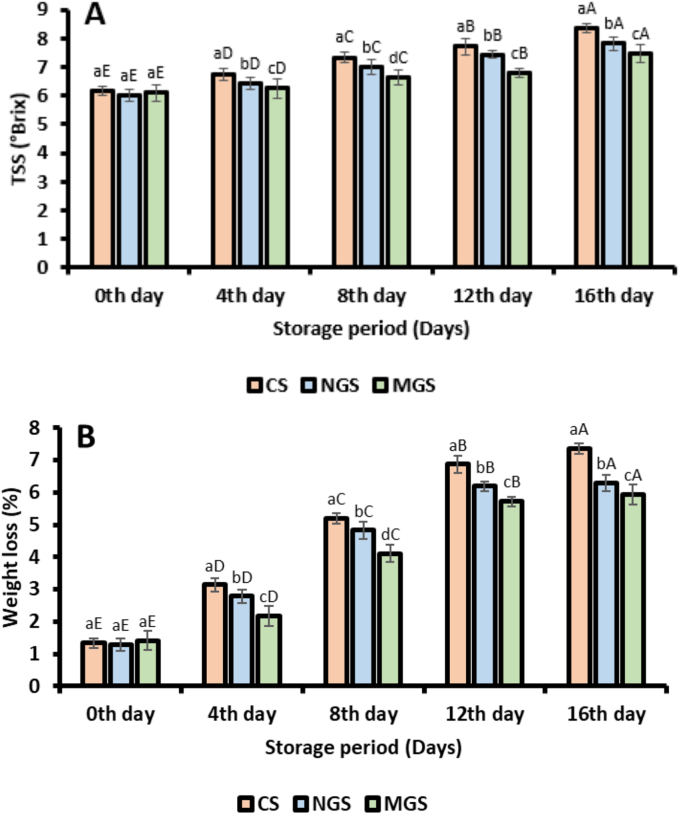

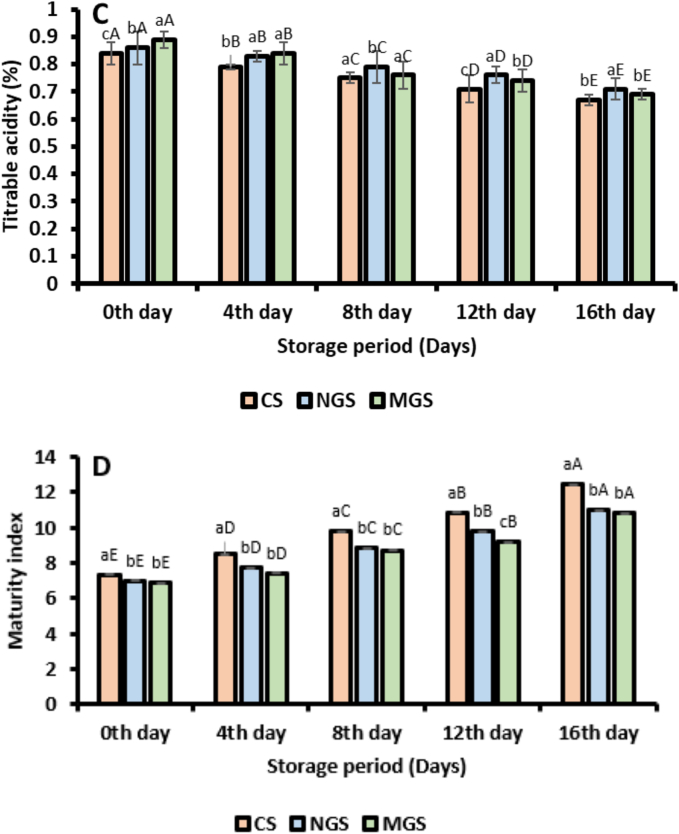

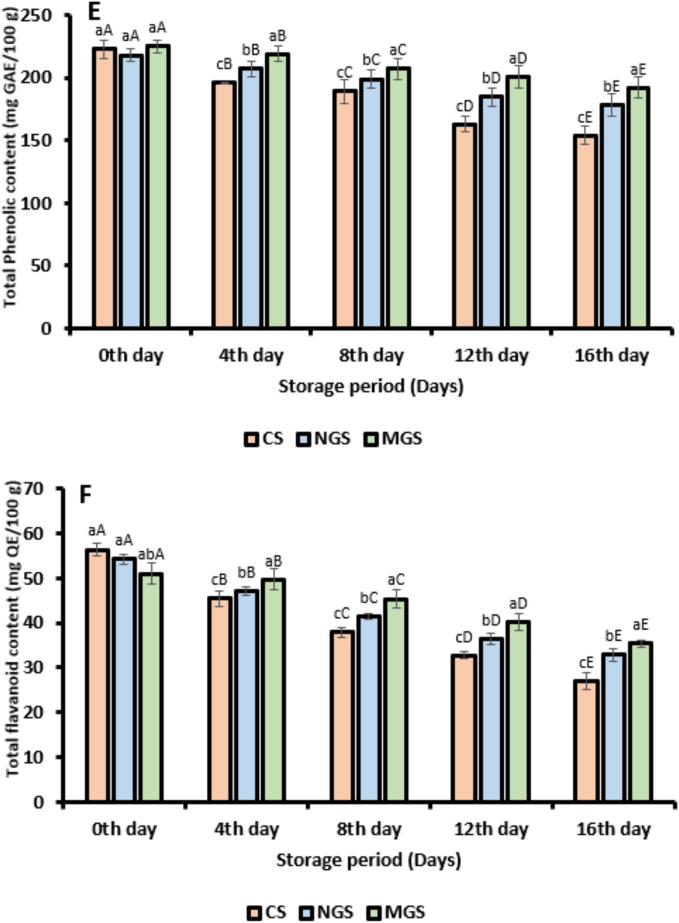

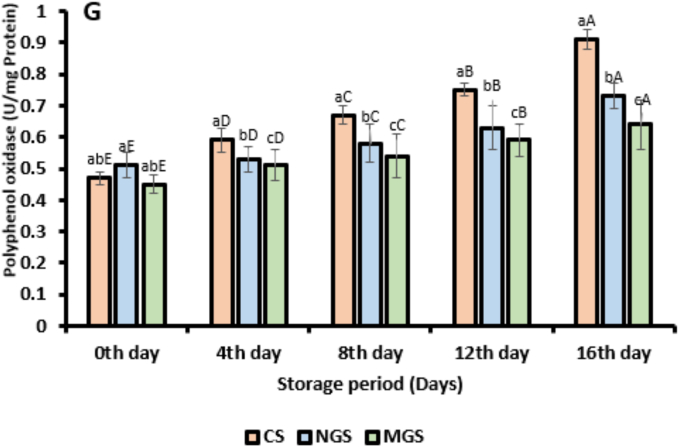
Physiochemical analysis of coated and uncoated strawberry. (A) Total soluble solids (TSS), (B) weight loss, (C) titrable acidity, (D) maturity index, (E) TPC, (F) TFC, (G) Polyphenol oxidase activity (CS; uncoated strawberry, NGS; strawberry coated with native gum arabic, MGS; strawberry coated with citric acid modified gum arabic) (Different error bars represent the standard deviation from the mean values (*n* = 3) and lowercase (a–c) or uppercase (A-E) represent the significantly different from each other).

The effect of coating treatments on the retention of total flavonoid content (TFC) in strawberries over a 16-day storage period is presented in Fig. 4F. All three formulations utilized control (CS), native gum arabic-coated (NGS), and citric acid-modified gum arabic-coated strawberries (MGS) exhibited a gradual decline in TFC during storage. However, the extent of flavonoid degradation varied significantly among treatments. At day 0, the TFC values of CS, NGS, and MGS were 56.33 ± 0.18, 54.19 ± 0.31, and 51.03 ± 0.20 mg quercetin equivalents (QE) per 100 g fresh weight, respectively. Despite MGS having the lowest initial TFC, it demonstrated superior retention of flavonoids over time. By day 16, MGS retained 35.35 ± 0.43 mg QE/100 g FW (30.71 ± 0.36 %). The significantly higher retention of TFC in MGS-treated fruits can be attributed to the synergistic action of citric acid and gum arabic in the coating matrix. Citric acid is known to inhibit polyphenol oxidase and protect flavonoids by stabilizing their structure under oxidative stress. Furthermore, its presence lowers the pH at the fruit surface, suppressing enzymatic activity responsible for flavonoid degradation (Carbone et al., 2009). Gum arabic provides an effective barrier against oxygen and moisture, reducing oxidative stress.

#### 3.7.5 Polyphenol oxidase (PPO)

The activity of polyphenol oxidase (PPO) in coated and uncoated strawberries during 16 days of storage is shown in Fig. 4G. PPO activity showed an increasing trend across all treatments as storage progressed, with significant differences observed among the control (CS), native gum arabic-coated (NGS), and citric acid-modified gum arabic-coated (MGS) samples. On day 0, PPO activity ranged from 0.45 ± 0.06 U/mg protein (MGS) to 0.51 ± 0.09 U/mg protein (NGS), indicating minimal variation at the start of storage. However, as storage time increased, PPO activity increased in the uncoated control sample, reaching 0.91 ± 0.05 U/mg protein by day 16. This substantial increase reflects typical enzymatic browning processes in strawberries due to phenolic oxidation under aerobic conditions and the absence of a protective barrier. In contrast, coated samples exhibited attenuated increases in PPO activity. The NGS treatment (native gum arabic) showed moderate PPO inhibition, rising to only 0.73 ± 0.15 U/mg protein at day 16. The MGS formulation demonstrated the strongest inhibitory effect on PPO activity, recording the lowest value (0.64 U/mg protein) at the end of the storage period. The superior performance of the MGS coating can be attributed to the presence of citric acid, a known PPO inhibitor. Citric acid acts by chelating the copper ions at the active site of the enzyme and lowering the pH at the fruit surface, thereby reducing enzymatic activity (Behera et al., 2021). Additionally, the citric acid modification of gum arabic enhances its barrier properties, limiting oxygen diffusion required for PPO-mediated oxidation reactions. These physicochemical properties effectively delay enzymatic browning and phenolic degradation during storage (Hamdan et al., 2022).

#### 3.7.6 Color value

The color attributes of strawberries, represented by L* (lightness), a* (red-green), and b* (yellow-blue) values, underwent significant changes over the 16-day storage period, with notable differences observed among the three treatments: uncoated strawberries (CS), native gum Arabic-coated strawberries (NGS), and citric acid-modified gum Arabic-coated strawberries (MGS). The L* value, indicating brightness, declined steadily across all treatments, with the uncoated strawberries showing the most pronounced reduction from 35.50 ± 0.39 on Day 0 to 23.40 ± 0.25 on Day 16 (Table 2C). This rapid darkening is attributed to higher moisture loss, tissue breakdown, and pigment degradation in the absence of a protective barrier (Khodaei et al., 2021). Coated samples, particularly those treated with MGS, retained higher L* values, with MGS exhibiting better brightness retention (26.35 ± 0.37 on Day 16) compared to NGS (27.29 ± 0.30). The citric acid-modified gum Arabic coating provided a more effective barrier, reducing moisture loss and oxidative reactions, thereby preserving lightness. The a* value, reflecting redness, also decreased over time, with the uncoated samples experiencing the steepest decline from 16.47 ± 0.45 to 10.43 ± 0.28 by Day 16. This is likely due to faster oxidative degradation and enzymatic browning. In contrast, NGS and MGS samples retained higher redness levels, with final a* values of 12.44 ± 0.40 and 12.69 ± 0.46, respectively. The superior retention of redness in MGS-treated strawberries can be attributed to the antioxidant properties and enhanced film-forming ability of citric acid-modified gum Arabic, which effectively mitigated pigment oxidation. Similarly, the b* value, which represents yellowness, showed a steady decline during storage, with uncoated samples decreasing from 23.05 ± 0.35 to 20.10 ± 0.20. Coated samples exhibited slower reductions in b* values, with MGS-treated strawberries showing the most gradual decline to 18.82 ± 0.39 by Day 16, compared to 18.60 ± 0.25 in NGS. This indicates that MGS provided better protection against the degradation of yellow pigments and reduced browning reactions. Overall, the decline in L*, a*, and b* values reflect the natural ripening and degradation processes in strawberries during storage. The application of citric acid-modified gum Arabic (MGS) significantly slowed these changes by forming a protective barrier that minimized moisture loss, oxidative stress, and enzymatic activity.

#### 3.7.7 Microbial analysis

The microbial quality of strawberries during storage was evaluated by monitoring yeast and mold count (YMC) and total plate count (TPC) over a 16-day period for three treatments: uncoated strawberries (CS), native gum Arabic-coated strawberries (NGS), and citric acid-modified gum Arabic-coated strawberries (MGS). Yeast and mold were not detected (ND) in any of the treatments during the initial days (0th and 4th). By the 8th day, YMC was first observed in the uncoated samples (CS) at 0.09 ± 0.18 log CFU/g, whereas no growth was detected in NGS or MGS (Table 2B). On the 12th day, YMC increased to 0.18 ± 0.09 log CFU/g in CS, while NGS and MGS exhibited lower counts of 0.14 ± 0.04 and 0.11 ± 0.13 log CFU/g, respectively. By the 16th day, YMC reached 0.24 ± 0.18 log CFU/g in CS, with comparatively lower counts in NGS (0.19 ± 0.13 log CFU/g) and MGS (0.15 ± 0.06 log CFU/g). Similarly, TPC followed a comparable trend. Bacterial growth was not detected during the initial storage days. By the 8th day, TPC reached 0.14 ± 0.15 log CFU/g in CS, while NGS and MGS continued to show no detectable levels. On the 12th day, TPC increased to 0.23 ± 0.19 log CFU/g in CS, with lower counts observed in NGS (0.19 ± 0.11 log CFU/g) and MGS (0.14 ± 0.07 log CFU/g). By the 16th day, the highest TPC was observed in CS (0.38 ± 0.09 log CFU/g), followed by NGS (0.31 ± 0.12 log CFU/g) and MGS (0.27 ± 0.14 log CFU/g). The reduced microbial counts in NGS and MGS samples highlight the effectiveness of edible coatings in mitigating microbial growth. (Guo et al., 2018). The superior performance of MGS can be attributed to its enhanced barrier properties, which minimize oxygen and moisture exchange, critical factors for microbial proliferation. Additionally, the citric acid modification of gum Arabic in MGS likely imparts antimicrobial properties, further inhibiting microbial growth. In contrast, uncoated strawberries (CS) exhibited the highest microbial counts due to greater exposure to oxygen, moisture, and external contaminants.

**Table 2B:** Color value of native and selected organic acid-modified gum coated strawberry.

Parameter	Samples	Storage period (Days)				
		0th day	4th day	8th day	12th day	16th day
L* value	CS	35.50 ± 0.39^aA^	33.10 ± 0.14^bB^	29.40 ± 0.53^cB^	26.70 ± 0.45^dB^	23.40 ± 0.25^eB^
	NGS	35.83 ± 0.14^aA^	34.17 ± 0.21^bA^	31.13 ± 0.27^cA^	29.29 ± 0.27^dA^	27.29 ± 0.30^eA^
	MGS	35.76 ± 0.18^aA^	34.67 ± 0.29^bA^	31.59 ± 0.40^cA^	29.41 ± 0.23^dA^	26.35 ± 0.37^eA^
a* value	CS	16.47 ± 0.45^aA^	14.50 ± 0.16^bB^	12.21 ± 0.19^cB^	11.66 ± 0.43^dB^	10.43 ± 0.28^eB^
	NGS	16.20 ± 0.23^aA^	15.38 ± 0.39^bA^	13.34 ± 0.22^cA^	12.39 ± 0.25^dA^	12.44 ± 0.40^dA^
	MGS	16.28 ± 0.24^aA^	15.81 ± 0.16^bA^	13.43 ± 0.34^cA^	12.53 ± 0.12^dA^	12.69 ± 0.46^dA^
b* value	CS	23.05 ± 0.35^aA^	22.70 ± 0.30^bB^	21.08 ± 0.25^cB^	20.50 ± 0.25^dA^	20.10 ± 0.20^eA^
	NGS	23.49 ± 0.30^aA^	22.50 ± 0.25^bA^	21.80 ± 0.20^cA^	21.40 ± 0.20^dA^	18.60 ± 0.25^eB^
	MGS	23.09 ± 0.29^aA^	22.69 ± 0.44^bA^	21.95 ± 0.38^cA^	21.87 ± 0.49^dA^	18.82 ± 0.39^eB^

The results were expressed as the mean ± standard deviation of ≥3 independent replicates and error bars represent the standard deviation from the mean values. In contrast, different lowercase (a–e) and uppercase (A–B) letters above each bar represent significantly different values within the storage time (days) and treatments, respectively, based on analysis of variance (ANOVA) and post hoc tests. (CS; uncoated strawberry, NGS; strawberry coated with native gum arabic, MGS; strawberry coated with citric acid modified gum arabic).

**Table 2C:** Microbial analysis of coated and uncoated strawberry.

Parameter	Samples	Storage days				
		0th day	4th day	8th day	12th day	16th day
YMC (log CFU/g)	CS	ND	ND	0.09 ± 0.18^bC^	0.18 ± 0.09^bB^	0.24 ± 0.18^dA^
	NGS	ND	ND	ND	0.14 ± 0.04^c^	0.19 ± 0.13^e^
	MGS	ND	ND	ND	0.11 ± 0.13^d^	0.15 ± 0.06^f^
TPC (log CFU/g)	CS	ND	ND	0.14 ± 0.15^a^	0.23 ± 0.19^a^	0.38 ± 0.09^a^
	NGS	ND	ND	ND	0.19 ± 0.11^b^	0.31 ± 0.12^b^
	MGS	ND	ND	ND	0.14 ± 0.07^c^	0.27 ± 0.14^c^

YMC, Yeast and Mold Count; TPC, Total Plate Count; ND, not detected. *Data is presented as.

mean ± SD (n = 3). Mean values within a column with different uppercase superscripts (A and.

B) and row with different lowercase superscripts (a and b) are significantly different (*p* < 0.05) from each other. CFU, colony-forming unit. (CS; uncoated strawberry, NGS; strawberry coated with native gum arabic, MGS; strawberry coated with citric acid modified gum arabic).

## 4 Conclusion

This study demonstrates the significant impact of organic acid modification on the structure and functional properties of GA and GG particularly in the formulation of an edible coating for enhancing strawberry shelf life. The incorporation of organic acids such as CA, MA, and TA successfully modified the structural characteristics of the gums, resulting in nano-sized particles and enhanced functional properties. SEM and FTIR analyses confirmed the successful cross-linking of the gums with organic acids, leading to the formation of spherical to semi-spherical particles with increased structural complexity and stability. TGA further corroborated the enhanced stability of the modified gums, suggesting improved resistance to heat degradation, which is critical for maintaining the integrity of edible coatings during food storage. The application of the modified gum coatings on strawberries demonstrated their effectiveness in preserving the fruit's color, reducing weight loss, and controlling microbial load. Additionally, the reduction in titratable acidity indicated that the coating also helped in preserving the fruit's sensory qualities. Further research could optimize the modification process parameters and explore the impact of different organic acids to enhance gum functionality while minimizing adverse effects. Moreover, there has been limited research conducted on the modification of gums using organic acids to enhance their functional properties.

## CRediT authorship contribution statement

**Anchal Choudhary:** Writing – review & editing, Writing – original draft, Methodology, Investigation. **Mansuri M. Tosif:** Writing – review & editing, Writing – original draft, Investigation. **Aarti Bains:** Methodology, Formal analysis, Data curation, Conceptualization. **Gulden Goksen:** Validation, Supervision. **Rupak Nagraik:** Writing – original draft, Visualization, Validation. **Sanju Bala Dhull:** Software, Resources, Data curation. **Nemat Ali:** Validation, Funding acquisition. **Nazish Muzaffar:** Visualization, Funding acquisition. **Prince Chawla:** Visualization, Validation, Supervision.

## Funding

The authors are thankful to researchers supporting Project number (RSPD2025R940), King Saud University, Riyadh, Saudi Arabia for financial support.

## Declaration of competing interest

The authors declare that they have no known competing financial interests or personal relationships that could have appeared to influence the work reported in this paper.

## Data Availability

Data will be made available on request.
